# A comparative analysis of converters of tabular data into image for the classification of Arboviruses using Convolutional Neural Networks

**DOI:** 10.1371/journal.pone.0295598

**Published:** 2023-12-08

**Authors:** Leonides Medeiros Neto, Sebastião Rogerio da Silva Neto, Patricia Takako Endo

**Affiliations:** Universidade de Pernambuco, Recife, Pernambuco, Brazil; Newcastle University, UNITED KINGDOM

## Abstract

Tabular data is commonly used in business and literature and can be analyzed using tree-based Machine Learning (ML) algorithms to extract meaningful information. Deep Learning (DL) excels in data such as image, sound, and text, but it is less frequently utilized with tabular data. However, it is possible to use tools to convert tabular data into images for use with Convolutional Neural Networks (CNNs) which are powerful DL models for image classification. The goal of this work is to compare the performance of converters for tabular data into images, select the best one, optimize a CNN using random search, and compare it with an optimized ML algorithm, the XGBoost. Results show that even a basic CNN, with only 1 convolutional layer, can reach comparable metrics to the XGBoost, which was trained on the original tabular data and optimized with grid search and feature selection. However, further optimization of the CNN with random search did not significantly improve its performance.

## Introduction

Advances in information technology have enhanced data collection and storage methods, allowing the extraction of massive volumes of data that may become too large for conventional systems to manage [1]. Consequently, it is necessary to use suitable technology to extract relevant information. There is a variety of data types including: tabular data, text, images, and sound.

Tabular data has been cited as the most common type of data in literature and industry [2, 3]. It is used with Artificial Intelligence (AI) to enhance products and services [2]. AI refers to the use of techniques to model intelligent behavior [4], and includes supervised Machine Learning (ML), which can learn from past data (examples) to perform tasks such as predictions or classifications [5].

In supervised classification problems, models learn by training on data that has already been classified with respect to a target variable [6]. In the healthcare field, for example, data such as medical records can be a useful resource for training ML models to predict whether a patient is ill, such as in [7–9].

There are different techniques of ML such as Support Vector Machines, tree-based, and distance-based algorithms [10, 11]. In addition, there is a branch of ML that has been highlighted recently called Deep Learning (DL) [12]. DL models work by mimicking the inner workings of the human brain [13], utilizing the concept of artificial neural networks [12], and in some cases DL models perform better than ML [14]. DL models are extensively used with data such as images, sound, time series, text, signals, and sequences [15, 16]. Some DL models stand out, achieving high performance in image classification [17]. However, the use of DL with tabular data is not a common study subject [2].

One way to use DL with tabular data is by converting the data into images. For that, there are a few converters available currently [18, 19]. Some converters assign one pixel in the image to represent each feature of the data set, but the process in which the pixels are arranged may vary among converters. For example, the converter proposed by Sharma et al. [18] assigns a grayscale pixel for each feature according to its coordinates in a reduced 2D space, the color intensity represents feature value. The converter proposed by Zhu et al. [19] assigns pixels according to the distance metric in a grayscale image. Therefore, features that have a shorter distance will be closer in the image generated. Similarly, Bazgir et al. [20] uses a combination of distance metric and dimensionality reduction to assign grayscale pixels for each feature.

This work aims to compare the performances of different converters of tabular data into images by training and evaluating the performance of Convolutional Neural Network (CNN) models when classifying diseases (Arboviruses). We also want to determine whether a CNN model is better than the traditional ML in healthcare data sets.

To achieve the main goal of this work, the following specific objectives are required: enumerate and study the converters of tabular data into image, propose a methodology to select the best converter, analyze the performance of CNN models trained on images generated by the conversion of tabular data, and the ML model trained on the original data.

## Related works

We have found few related studies. Borisov et al. [2] presented a survey on the use of DL with tabular data. Some methods for using DL with tabular data include: specialized DL models, and data encoding techniques, one of which involves converting the tabular data into images. The authors argued that tabular data is heterogeneous in nature and the transformation into images makes the data homogeneous, which can improve the performance of CNN models [2].

The CNN model effectively learns patterns in homogeneous data, as observed by Borisov et al. [2]. Despite concerns about the potential reduction in heterogeneity between classes, the transformed data preserves enough differences to train CNN that perform better than ML models [18–20]. For example, in a related study, Sharma et al. [18] demonstrated superior performance of a CNN model compared to ML algorithms across multiple data sets by initially converting the data into images. This suggests that the conversion of tabular data into images might yield beneficial results.

Sharma et al. [18] introduced a conversion approach that maps features of a tabular data set to a 2D pixel grid by reducing the dimensionality of the data. The authors applied this method to gene expression, text, vowels, and two artificial data sets and found that a CNN model trained on the resulting images outperformed tree-based ML algorithms trained on the original data for all data sets.

Bazgir et al. [20] proposed a method called REpresentation of Features as Images with NEighborhood Dependencies (REFINED), that transforms tabular data into images using the Bayesian Multidimensional Scaling (BMDS) technique. It was applied to predict drug sensitivity in cancer cells. When compared to various ML algorithms the REFINED-CNN achieved improved accuracy. Furthermore, it also performed better than specialized architectures for predicting drug sensitivity in cancer cells [20].

Zhu et al. [19] presented a technique to convert tabular data into images called Image Generator for Tabular Data (IGTD). The technique assigns one pixel in a gray scale image for each feature, so similar features are placed closer together. As an example of applicability, the authors converted a data set composed of molecular descriptors of gene expression profiles of cancer cell lines, and molecular descriptors of drugs to predict anticancer drug response. Their experiments showed that the CNNs trained on images generated by the IGTD perform better than the CNN models trained on images from other converters and the ML models trained on the original tabular data.

## Background

This section presents the background necessary to better understand this work. Subsection Convolutional Neural Network with tabular data describes how CNN works and explains that it can be trained with images converted from tabular data, and use spatial information for improved classification.

Subsections DeepInsight, REpresentation of Features as Images with NEighborhood Dependencies (REFINED), and Image Generator for Tabular Data (IGTD) explain the converters for tabular data into image used in this work. Lastly, Subsection Metrics presents the evaluation metrics used in this work.

### Convolutional Neural Network with tabular data

When studying the visual system of cats, Hubel and Wiesel [21] discovered that some neurons activate in the presence of specific visual stimuli that increase complexity along the neural pathway, from edges, lines, and circles, to parts of the object, to the entire object [22]. This idea is brought to the CNN model which detects basic features in the first layers, that are then combined by higher layers, building a feature extractor [23], and then feed the features to a neural network.

A CNN is trained to recognize objects based on the spatial relationship between image pixels with the layers that implement the convolution operation. In pictures of real world objects this relationship is naturally present, for instance, the form or structure of a human head might differ, but some features remain the same [19]. This make CNN models excel at 2D image classification, but raw tabular data is still a challenge because of the format and lack of spatial information [2].

Nonetheless, it is possible to convert the data into images to leverage the power of 2D CNNs. However, an image composed of tabular data is not a real world object and thus contains no spatial coherence. To solve this issue, some works proposed representing the relationship between features as a spatial relationship between pixels by arranging them according to feature similarity [18–20]. The CNN models can pick up this spatial information to make classifications.

### DeepInsight

**Fig 1:**
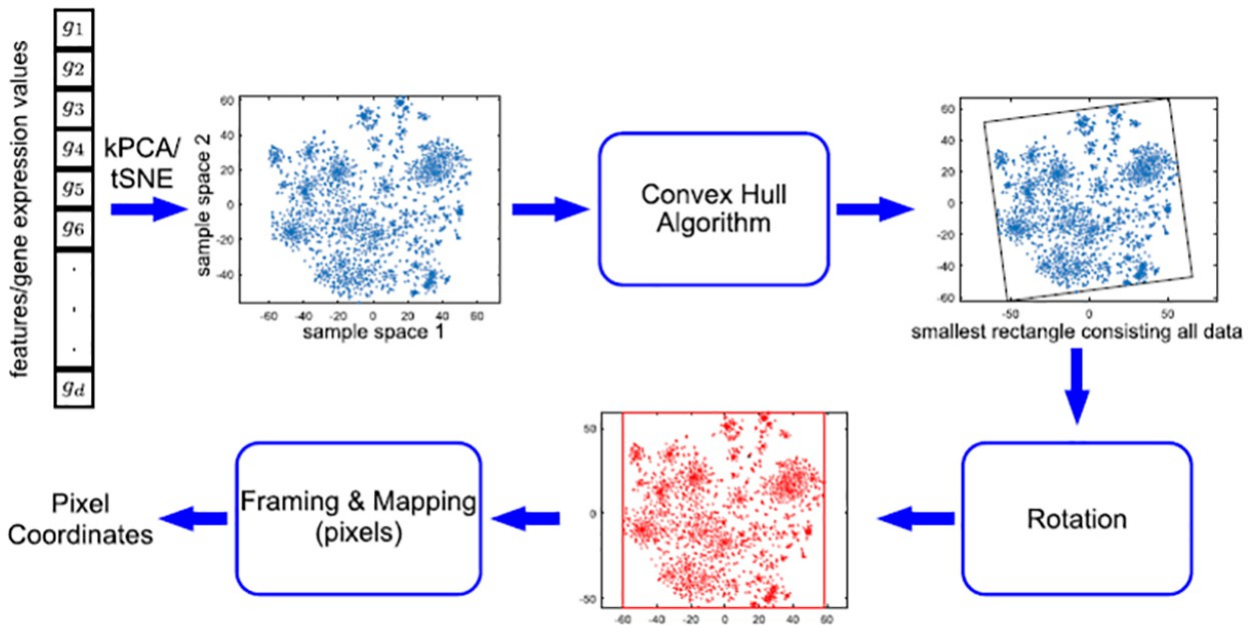
The process of conversion of the DeepInsight. Source: Sharma et al. [18].

The DeepInsight converter, proposed by Sharma et al. [18], assigns one pixel in a gray scale image for each feature, the color intensity represents the values, and it represents feature relation as spacial relation in the images by using an algorithm for dimensionality reduction. The process is shown in Fig 1, first, the DeepInsight reduces the dimensionality of the features to a 2D space using one of the available algorithms: T-Distributed Stochastic Neighbor Embedding (T-SNE), Principal Component Analysis (PCA), Kernel Principal Component Analysis (kPCA), or a user-defined one.

Then, the DeepInsight detects the smallest rectangle that contains all feature points in the reduced 2D space with the convex hull algorithm [24] and makes a rotation, if necessary, to keep the image rectangular. The converter assigns a pixel for each feature based on the coordinates in the reduced 2D space by framing the features into a pixel grid and mapping the respective values, keeping the distances proportional.

The user can select the image size manually. However, a few things need to be considered: If the user sets a small image size with respect to the number of features, some features may be placed in the same pixel (feature overlap). In that case, the pixel value will be the arithmetic mean of the overlapping features.

More than one feature in the same pixel, denoted as feature overlap, will result in a value that was not present in the original data set. Therefore, the best case scenario is one without feature overlap. [18]. Large image sizes may decrease feature overlap, but may also increase computational cost. On the other hand, a smaller image size results in lower computing cost, but higher feature overlap.

### REpresentation of Features as Images with NEighborhood Dependencies (REFINED)

The REFINED technique, introduced by Bazgir et al. [20], enhances the utility of CNNs in predictive modeling tasks involving spatially uncorrelated data. This is achieved by converting high-dimensional feature vectors into compact grayscale images, each pixel symbolizing a feature, rendering them suitable for CNN analysis.

In essence, REFINED transforms intricate feature vectors into justified images for traditional CNN training. Rather than a direct mapping onto a 2D image matrix that could lose spatial correlations, it employs the Bayesian variant of metric Multidimensional Scaling, known as BMDS.

Starting with the Euclidean Distance matrix of features, this acts as a measure of distance. Subsequently, the Multidimensional Scaling (MDS) algorithm creates a 2D space with feature coordinates, forming an initial feature map. By employing BMDS, feature positions in a confined space are estimated, ensuring that each pixel accommodates just one feature. A hill climbing algorithm optimizes this configuration by minimizing distance differences among these new feature positions.

A distinct coordinate space for each feature is generated, forming the basis for creating an image for every sample. These images are then harnessed to train a CNN. The REFINED approach combines feature mapping, and optimization, to produce effective data representation for training CNNs for data analysis.

### Image Generator for Tabular Data (IGTD)

**Fig 2:**
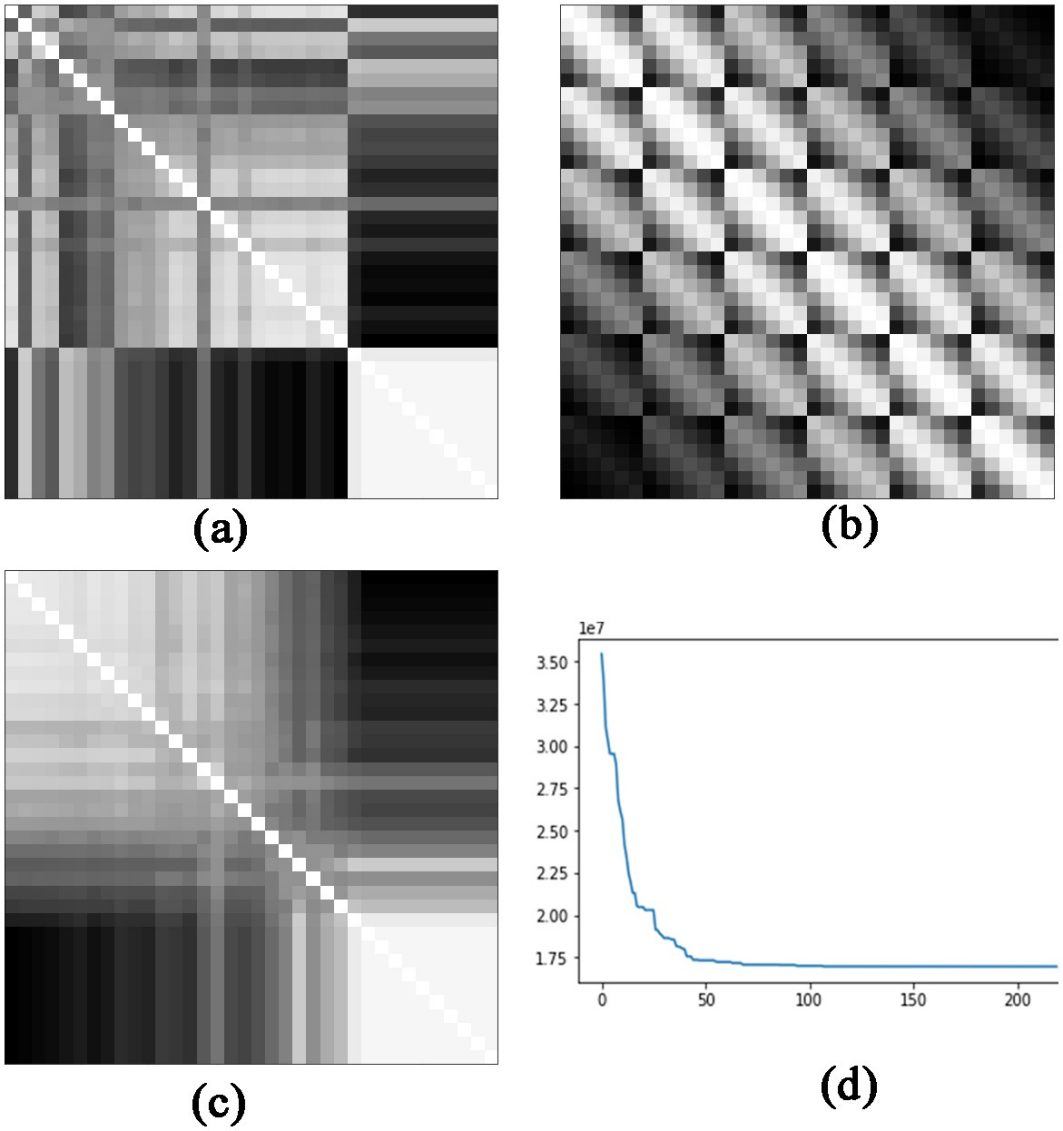
Example of distance matrices generated by the IGTD in the process of conversion. Examples of: (a) a feature distance ranking matrix using the Euclidean distance; (b) a pixel distance ranking matrix using the Euclidean distance; (c) a feature distance ranking matrix after optimization. The IGTD tries to minimize the error, that is, the difference between matrices (a) and (b).

Zhu et al. [19] proposed a converter of tabular data into images called IGTD that assigns one pixel in a gray scale image for each feature, the color intensity reflects the value of the feature. The IGTD represents the relation between features of the tabular data into relation between pixels in the image by using a distance metric. The converter calculates the distances of each feature to every other (pairwise distance) and attempts to assign pixels with similar distances in the image. It does this process by making use of the distance matrix of the features and pixels in ascending order (ranked), denominated the feature distance ranking matrix (Fig 2a), and the pixel distance ranking matrix (Fig 2b).

The IGTD optimizes the assignment of features by minimizing the differences between the two matrices. Because the 2D image is limited in space, the converter may not be able to keep the exact feature distance as pixel distance in the image, so an error function is defined to measure the differences between the feature distance and pixel distance ranking matrix, Fig 2c shows the error (y-axis) per number of iterations (x-axis).

As the number of iterations increases, the error decreases. At one point, the decrease in error might not be significant or stop altogether, so the user can set the algorithm to stop running at this point. Although it is common practice to use square images with CNNs [25], the IGTD might not produce square images. If necessary, zero-padding can be added to create square images [26].

### Metrics

Evaluation metrics come with different purposes and make different measurements. This work utilizes the Accuracy, Precision, Recall, F1 score, and Specificity metrics explained below.

#### Confusion matrix

True Positive (TP) are the elements that are positive in reality and were correctly identified by the model. False Positive (FP) are the elements that are negative in reality and were incorrectly labelled as positive by the model. Similarly, True Negative (TN) are negative elements correctly identified by the model and False Negative (FN) are positive elements incorrectly labeled as negative by the model [27]. These variables compose the confusion matrix, which is a matrix composed of the number of predicted positive/negative and actual positive/negative in different axis, the main diagonal consists of TP and TN [28]. The metrics are calculated based on this matrix.

#### Accuracy

Accuracy measures the correctly classified samples divided by both the correctly and incorrectly classified samples. The Accuracy is calculated by the following equation [28]:

$$\begin{array}{c} Accuracy=\frac{TP+TN}{TP+TN+FP+FN} \end{array}$$ (1)

#### Precision

Precision is a metric used to calculate the proportion of cases correctly classified as positive out of all the elements classified as positive by the model. It gives a measure of how well the model performs with respect to the positive cases, and is calculated by the following equation [28]:

$$\begin{array}{c} Precision=\frac{TP}{TP+FP} \end{array}$$ (2)

#### Recall

Recall, also known as sensitivity, represents the proportion of the positive cases in reality that were classified as positive by the model out of all the actual positive cases. Its equation is as follows [28]:

$$\begin{array}{c} Recall=\frac{TP}{TP+FN} \end{array}$$ (3)

#### F1-Score

When evaluating the performance of the models, it’s important to consider various metrics. In addition to the ones mentioned above, we also utilize the F1-Score. This metric is the harmonic mean between Recall and Precision, as presented in equation below [28]:

$$\begin{array}{c} F1-score=2\times \frac{Precision\times Recall}{Precision+Recall} \end{array}$$ (4)

#### Specificity

This metrics analyses the model’s performance with respect to the TN. Essentially, it represents how good the model is when it comes to classifying TNs [27]. Its equation is as follows [28]:

$$\begin{array}{c} Specificity=\frac{TN}{TN+FP} \end{array}$$ (5)

## Converting tabular data into images

This section describes the contributions and improvements that this work adds when converting tabular data into images. Section Conversion Quality Analysis for the DeepInsight describes the process proposed to improve the quality of the conversion with the DeepInsight [18].

### Conversion Quality Analysis for the DeepInsight

The DeepInsight converter, introduced by [18], allows for manual parameter selection, including the use of the Dimensionality Reduction (DR) algorithm and image size. However, it’s crucial to acknowledge that these choices cannot be arbitrary. When multiple features are assigned to the same pixel, resulting in feature overlap, the converter represents the arithmetic mean of those features in that pixel. This approach might yield values not present in the original data set, a limitation that might be unsuitable for certain real-world applications [18].

**Fig 3:**
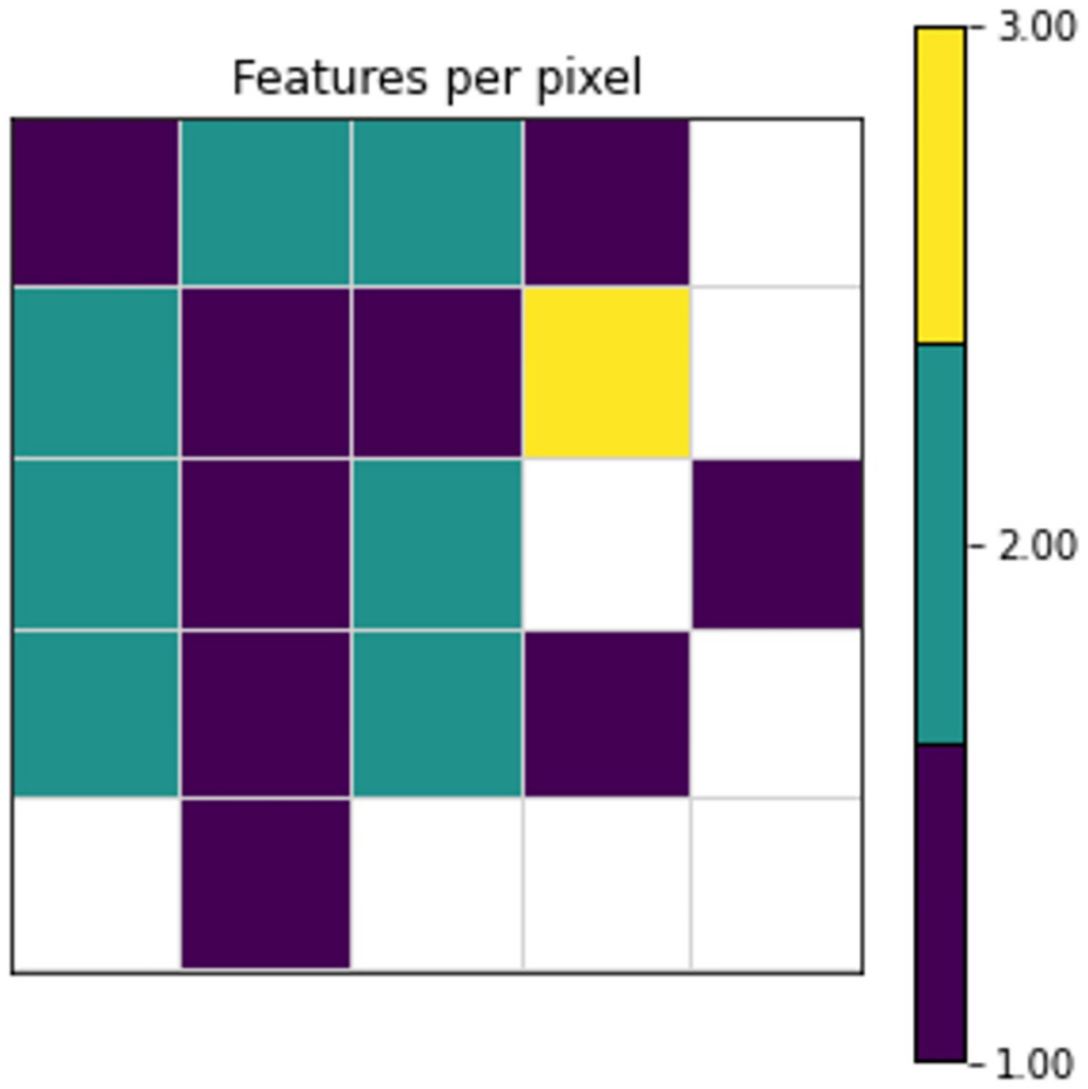
An example of a 5x5 Feature Density Matrix. Squares represent pixels, warmer color means more feature overlap.

Therefore, it is important to carefully analyze the parameter selection for DeepInsight, which can be done through the utilization of a resource called the Feature Density Matrix (FDM). The FDM offers insights into the extent of feature overlap or density, showing specific pixels with overlap occurrences. As depicted in Fig 3, an example of a 5x5 FDM generated using the T-SNE DR algorithm, one pixel holds the mean value of three features (highlighted in yellow), while seven pixels hold two features each (highlighted in green), and the remaining pixels contain one feature each, this scenario is not recommended.

**Fig 4:**
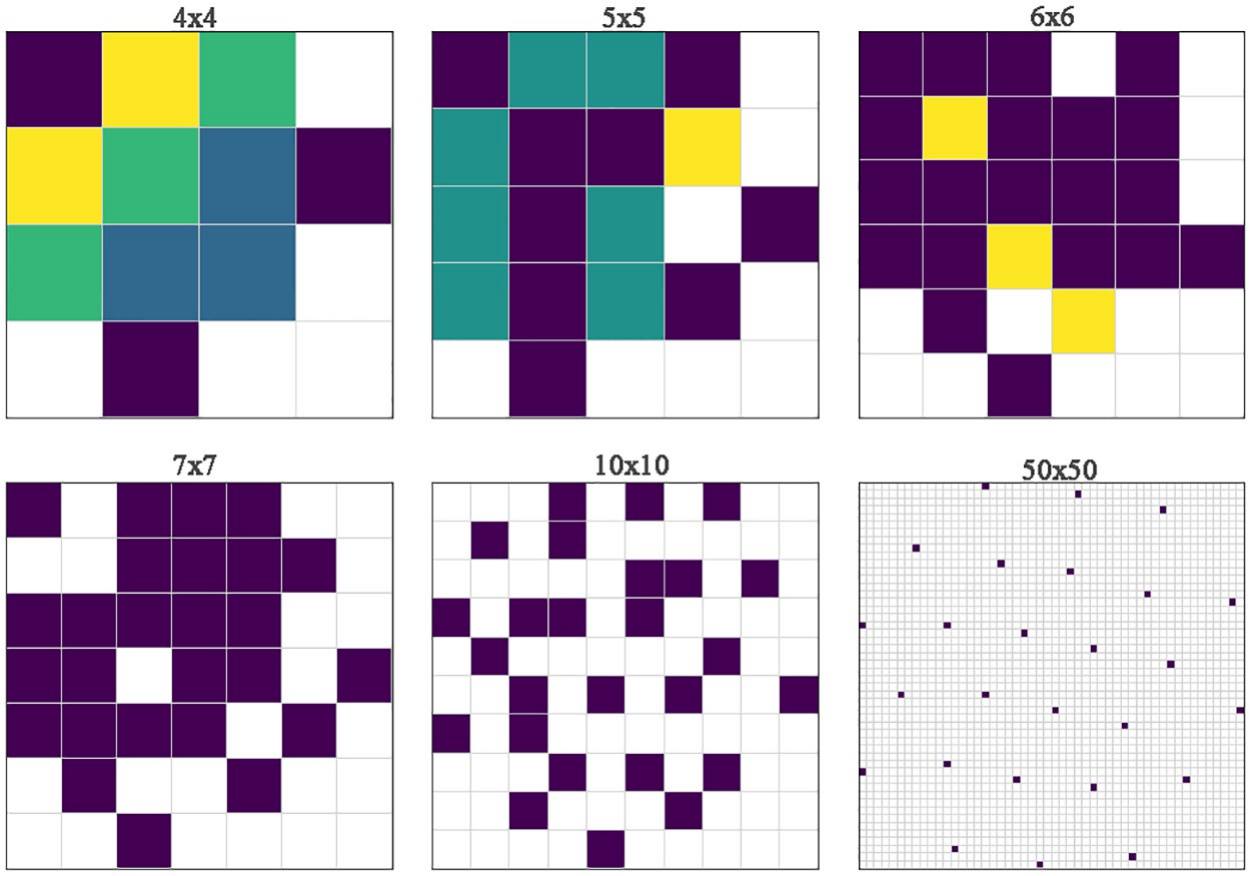
Image size vs feature overlap. As the user increases the image size, the feature overlap decreases. Each square represents a pixel, warmer color means more feature overlap.

To mitigate feature overlapping, users have the option to increase the image size. The relation between enlarging the image size and mitigating feature overlap is illustrated in Fig 4. In the case of the T-SNE DR algorithm, as depicted in the image, it’s evident that a minimum image size of 7x7 is required to eliminate feature overlap.

Determining the optimal size to achieve the least overlap for a specific data set while keeping the image small can be achieved by exhaustive exploration of various combinations of image sizes and DR algorithms. However, manual parameter search can be time-intensive, especially when dealing with large data sets.

Currently, to the best of our knowledge, there is no established numerical metric or systematic process for selecting the best combination of DeepInsight’s parameters. To address this gap, we propose a novel methodology, called the **Conversion Quality Analysis (CQA)**.

The objective is to determine the DR algorithm that minimizes the feature overlap while using the smallest image size within the user defined limits. In the CQA process, users need to determine the maximum image size they are willing to accept based on factors such as hardware specification, time constraints, and data set characteristics. This decision considers the worst-case scenario to ensure completion within available resources given that, in general, large image sizes utilize more computational power. In other words, users should answer the question: *In the worst-case scenario, what is the maximum image size I can afford to use?*

The next step involves considering a list of DR algorithms for selection. DeepInsight offers three predefined DR algorithms: T-SNE, PCA, and kPCA. Alternatively, users can also utilize custom DR algorithms as long as they implement a fit_transform method.

In order to determine the most suitable DR algorithm, we propose two metrics: *(i)* Percentage of Overlapped Features (OF) and *(ii)* Percentage of Overlapped Pixels (OP). OF represents the ratio of overlapped features to the total number of features, multiplied by 100, as shown in Eq 6:

$$\begin{array}{c} OF=(\frac{O}{F})\times 100 \end{array}$$ (6)

where O is the number of overlapped features and F is the total number of features.

Similarly, OP corresponds to the ratio of pixels containing feature overlap to the total number of pixels, multiplied by 100, as defined in Eq 7:

$$\begin{array}{c} OP=(\frac{O}{P})\times 100 \end{array}$$ (7)

where O is the number of pixels that contain feature overlap and P is the total number of pixels.

By utilizing the OF and OP metrics, the CQA aids in the identification of the combination of DR algorithm and image size that minimizes the feature overlap while utilizing the smallest possible image size within the defined constraints.

**Fig 5:**
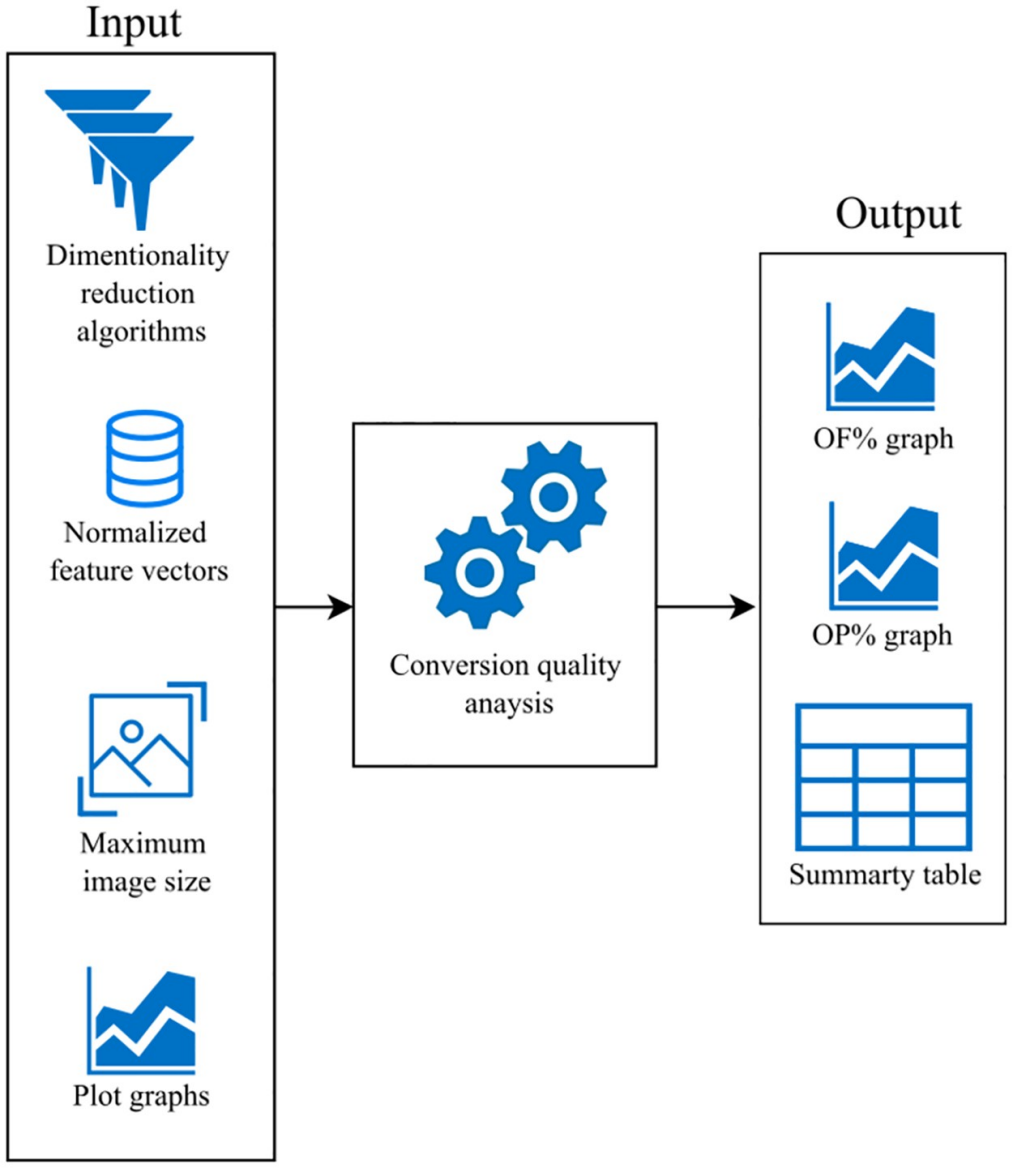
The CQA process.

We have implemented this process in a Python code [29], which primarily consists of a function that accepts normalized data set, the maximum allowable image size, and a list of DR algorithms as input. This function calculates the metrics that quantify the extent of feature and pixel overlap present in the matrix. The code iterates over the image sizes until the maximum allowed, generates the FDM, and computes the OF and OP metrics for each combination of DR and image size. The outputs are the OF% and OP% per image size graphs, and a summary table that presents the minimum feature and pixel overlap metrics for each DR. Fig 5 illustrates the process.

This summary table (Table 1) can be used to compare the performance of different DR and identify the one with the least overlap. The code also plots the percentage of overlapped features and pixels for each DR across image sizes. This systematic approach helps researchers select the optimal DR algorithm and image size for their data set.

**Table 1:** Example of a summary table.

	Min OP%	Min OP%	Matrix Size
TSNE	0.000000	0.000000	7x7
PCA	42.307692	0.036523	71x71
KernelPCA	42.307692	0.036523	71x71

## Classifying Arbovirus

Arboviruses are a group of diseases caused by arboviruses (arthropod-borne viruses), including Dengue (DENV), Chikungunya (CHIKV), Zika (ZIKV), and Yellow Fever. These diseases can cause health problems and even death in some cases, and are found all over the world, primarily in tropical areas [30].

*Aedes Aegypti* and *Aedes Albopictus* mosquitoes are the primary vectors of arboviruses. Population growth and disorderly migration, inadequate urbanization, irregular provision of health services, and increased population density are all factors that have contributed to their spread [27].

Despite government efforts to combat these mosquitos, there is still a greater challenge: accurate diagnosis of these arboviruses. DENV, CHIKV, and ZIKV diseases, for example, share symptoms such as fever, myalgia, arthralgia, and exanthema. Furthermore, it is possible that cross-reactions are presented by serological tests in the detection of these arboviruses [27, 31]. All of these factors make a differential and accurate diagnosis difficult.

### Arboviruses data sets

The data set used in this work is open to the public [32], and it was originally pre-processed by Tabosa de Oliveira et al. [7], they propose ML models for the multi-class classification of Arboviruses. The data is composed of clinical and sociodemographic data of patients for the classification of the classes Dengue, Chikungunya and Others.

The data set contains notifications of Dengue and Chikungunya from the State of Amazonas, Brazil, and from the City of Recife, Pernambuco, Brazil. The source of the Amazonas data is the Brazilian Information System for Notifiable Diseases (SINAN) from Portuguese: *Sistema de Informação de Agravos de Notificação*, and the source of the Recife data is the Open Data Portal of Recife [7]. The authors selected 27 features with the help of healthcare professionals, and preprocessed the data set.

This work focuses on two binary classifications: *(i)* Arboviruses and Inconclusive; and *(ii)* Dengue and Chikungunya. The Arboviruses data set was divided into two data sets, one for each classification problem, as shown in Table 2. For classification problem *(i)*, the data set is called Arboviruses/Inconclusive, and it is composed of the Arboviruses class with 2862 records of each disease (5724 total), and the Inconclusive class with 5724 records. For the classification problem *(ii)*, the data set was called Dengue/Chikungunya, and it is composed of 5724 records of each disease. The study of Da Silva Neto et al. [9] also utilizes the same data set with the same division as our work, they propose binary ML models with which we compare our models.

**Table 2:** Composition of the Arboviruses data sets.

Data set	Classes		Total	Features
Arboviruses/Inconclusive	Arboviruses	2862 Dengue cases; 2862 Chikungunya cases	11448	27
	Inconclusive	5724 Inconclusive cases		
Dengue/Chikungunya	Dengue	5724 Dengue cases	11448	
	Chikungunya	5724 Chikungunya cases		

### Experiment methodology

This work uses three converters for tabular data into images to train CNNs and compare their performances. The first converter is the DeepInsight, which is proposed by Sharma et al. [18], and available on Github [33] where there is a version for Matlab and Python, we used the Python version. The second converter is the REFINED, proposed by [20], available in [34]. Lastly, the IGTD proposed by Zhu et al. [19], in their work the authors provide a Github [35] with the code and examples.

**Fig 6:**
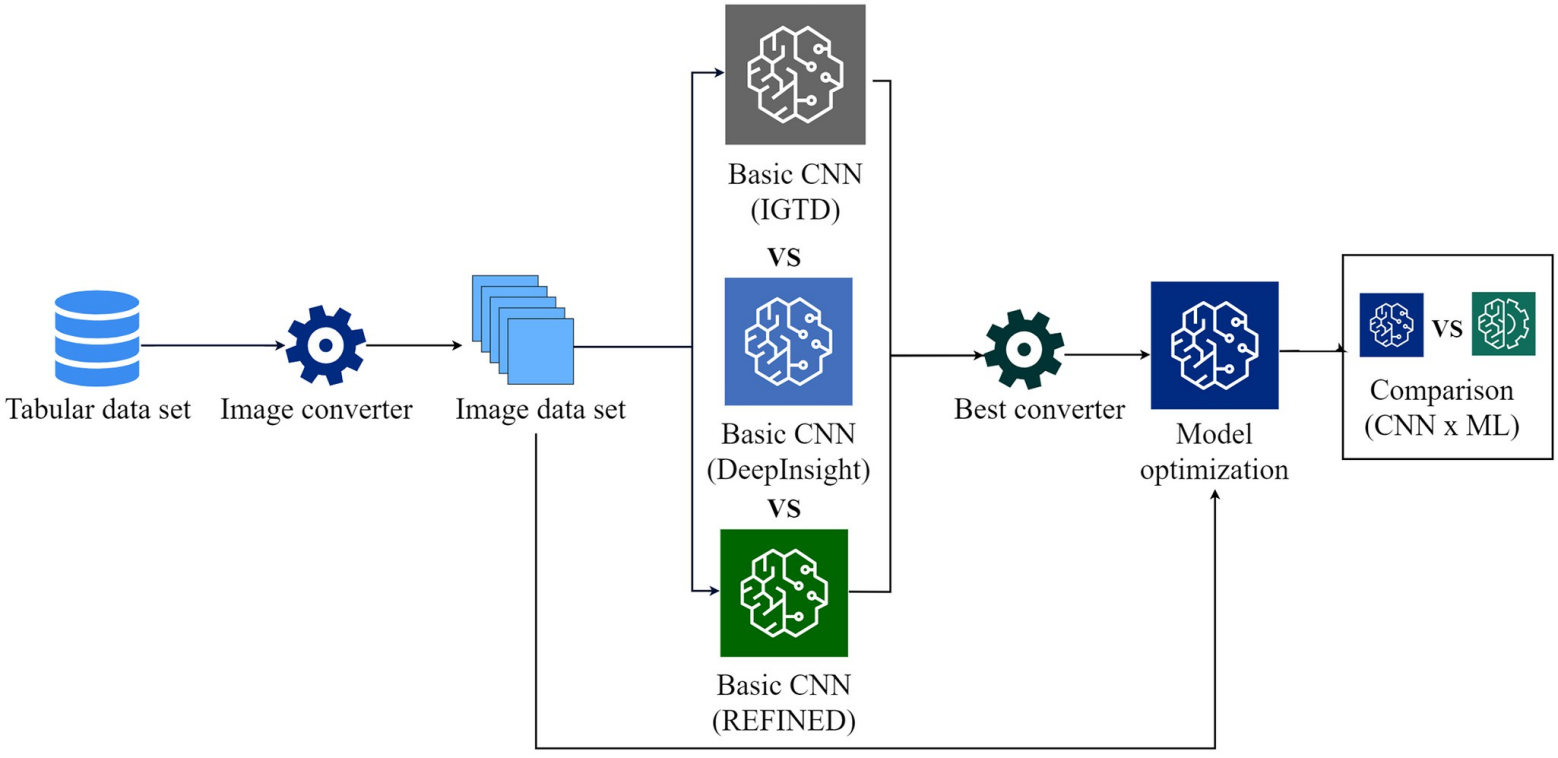
Experiment methodology.

The process by which we conducted the experiments is summarized in Fig 6. First, the tabular data sets are converted into images using the converters, the images are used to train Basic CNN models, one for each converter. We make a comparison between our basic models and choose the one with the highest evaluation metrics, then we fine tune a CNN model trained with the image data set of the best converter, and compare with the ML model trained on the original tabular data. To build and train the CNNs, we employed the Sequential model from Keras [36], a DL library based on TensorFlow [37]. The training process involved the following key steps: data loading, model compilation, training, and testing. We utilized the Scikit Learn library [38] for the evaluation metrics.

The ML model we chose was the eXtreme Gradient Boosting (XGBoost) which was the model that achieved highest metrics proposed by Da Silva Neto et al. [9], on the same classification problems with the same data set.

## Results

This section presents the results of the parameters used for the conversions, samples of the tabular data converted into images, and the performance results of the CNN models. Subsection IGTD parameters for the Arboviruses data sets presents the parameters used for the IGTD converter. Subsection DeepInsight parameters for the Arboviruses data sets presents the parameters used for the DeepInsight converter. Finally, subsection Results of the Basic CNNs presents the results of the experiments.

### DeepInsight parameters for the Arboviruses data sets

**Fig 7:**
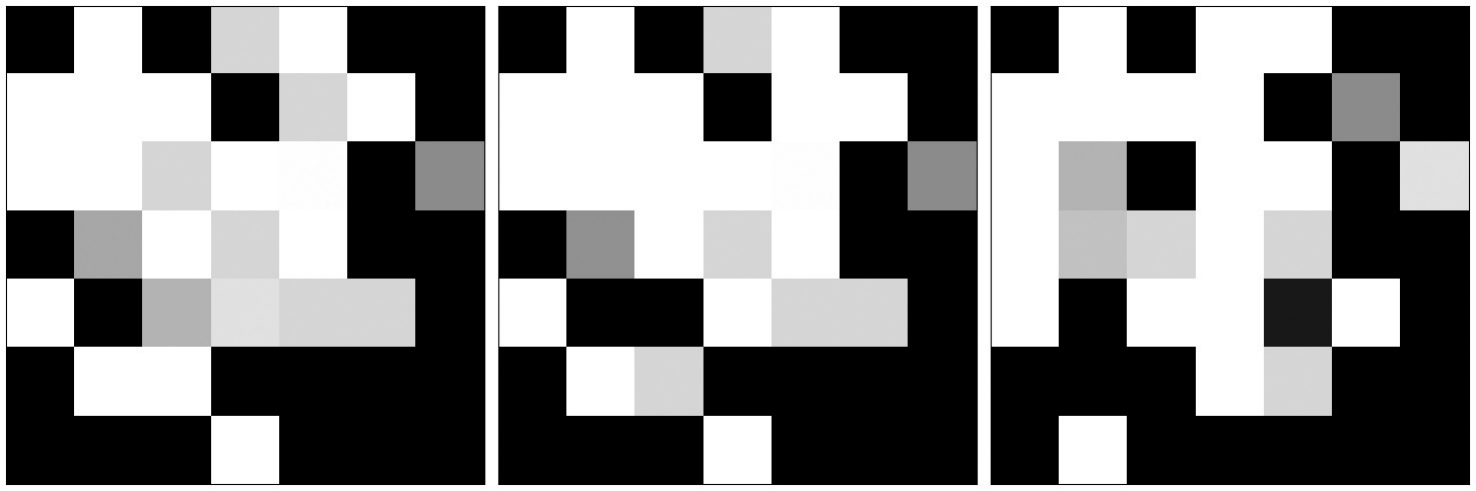
Samples of Dengue, Chikungunya and Inconclusive converted by the DeepInsight. Whiter color means greater feature value.

Table 3 shows the parameter configuration of the DeepInsight converter for the Arboviruses data sets. To choose the parameters, first, the tabular data set was normalized using the built-in logarithmic scaler as in Sharma et al. [18]. We analyzed the 3 dimensionality reduction techniques previously available by the converter, and we used the CQA process to choose one that produced the least feature overlap at the smallest image size: the T-SNE, with image size 7x7. We also defined a *random_state* of 1701 for reproducibility. Fig 7 shows some images samples of the Arboviruses data sets after the conversion.

**Table 3:** Parameter of the DeepInsight converter for the Arboviruses data sets.

Parameter	Value	Description
feature_extractor	t-sne	String (‘tsne’, ‘pca’, ‘kpca’) or a class with method ‘fit_transform’ that returns a 2-dimensional array of extracted features.
pixels	7	Int (square matrix) or tuple of ints (height, width) that defines the size of the image matrix.
random_state	1701	Determines the random number generator, if present, of a string defined feature_extractor.

### REFINED parameter configuration

**Fig 8:**
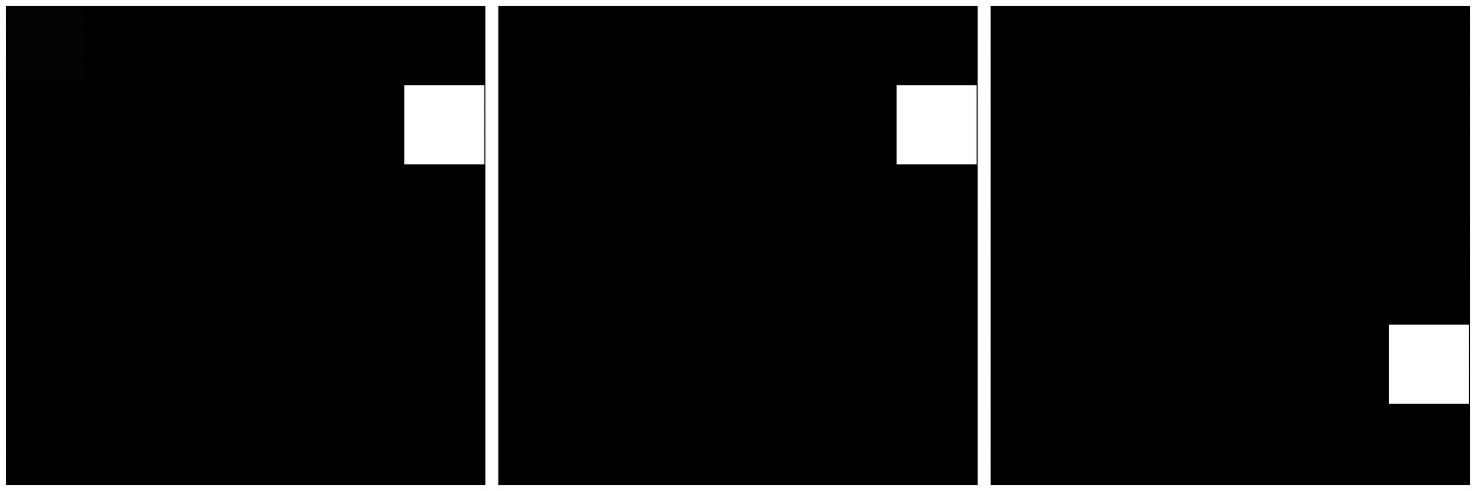
Samples of Dengue, Chukungunya, and Inconclusive cases, respectively, converted by the REFINED.

The REFINED algorithm requires a single parameter, which is the number of iterations. This parameter determines the number of times the hill climbing algorithm will go through all the features and check the cost of exchanging each feature. In essence, the number of iterations influences the extent to which the algorithm explores and evaluates different feature exchange possibilities. By adjusting this parameter, users can control the thoroughness of the hill climbing process in optimizing the feature exchange cost. We chose to use the same parameter value as the original work: 5. Fig 8 shows samples of Arbovirus data converted into images by the REFINED converter.

### IGTD parameters for the Arboviruses data sets

Table 4 shows the parameter configuration of the IGTD for the Arboviruses data sets. The first parameter, *norm_data*, is the tabular data normalized to convert into images. *num_row* and *num_colum* are the number of rows and columns in the images, these numbers must be set so that when multiplied they equal the total number of features. We added 10 zero-padding columns to produce 6x6 square images. The parameter *save_image_size* is the size in inches of the preview images that the algorithm generates, we found that 0.16 inches (0.41 cm) is enough to visualize them clearly. The next parameter, *max_step* is the maximum number of iterations to run the converter in case it does not converge, this serves as an early-stop mechanism. Algorithm convergence is determined by whether the error reduction rate is smaller than a threshold for the number of iterations of *val_step*.

**Table 4:** Parameter configuration of the IGTD converter for the Arboviruses data sets.

Parameter	Value	Description
norm_data	Data set normalized	Data set for the conversion
num_row	6	Number of pixel rows in image representation.
num_col	6	Number of pixel columns in image representation.
save_image_size	10	Size of preview pictures (in inches).
max_step	1000	Maximum number of iterations, if it does not converge.
val_step	100	Number of iterations for determining algorithm convergence.
fea_dist_method	Euclidian	Feature distribution method
image_dist_method	Euclidian	Image distribution method
error	Squared	Error function
switch_t	0	In each iteration, if the smallest error change rate resulted from all possible feature swapping is not larger than switch_t, the feature swapping that results in the smallest error change rate will be performed. If (*switch*_*t* < = 0), the IGTD algorithm monotonically reduces the error during optimization.
min_gain	0.00001	If the error reduction rate is not larger than min_gain for val_step iterations, the algorithm converges.

**Fig 9:**
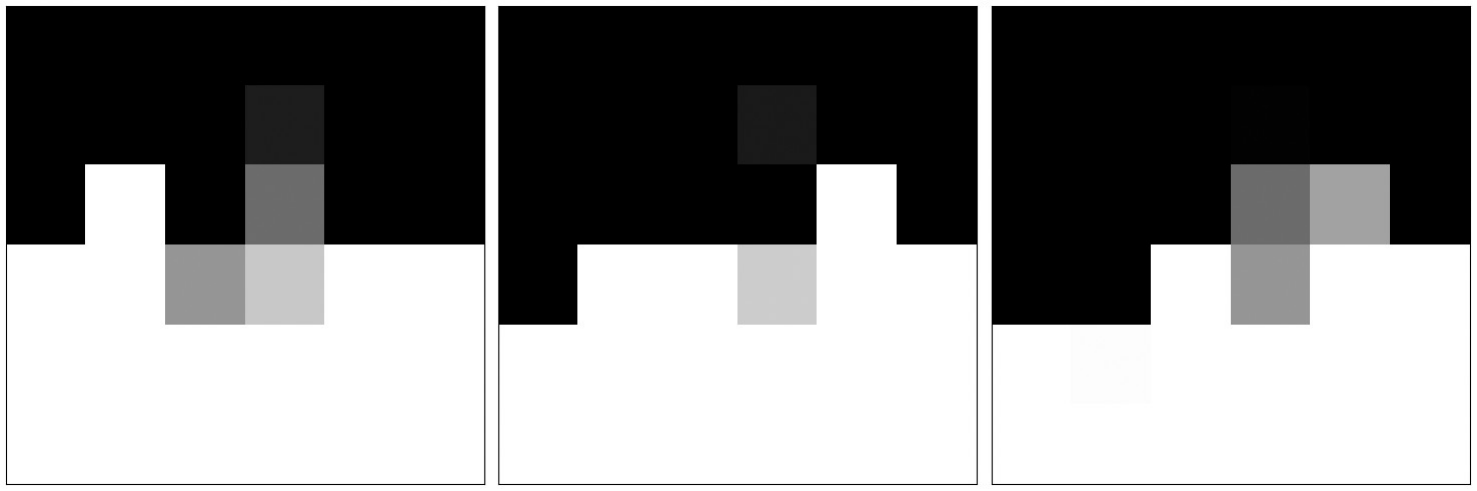
Samples of Dengue, Chukungunya and Inconclusive converted by the IGTD.

The parameter *fea_dist_method* determines which metric to use to calculate feature distance, similarly, *image_dist_method* is the metric to calculate the pixel distances. The parameter *error* determines the error function to use. When arranging the pixel locations for each feature, the algorithm considers all feature swaps and the change in error that they might produce, if this change is not larger than *switch_t* the feature swap is performed. Finally, *min_gain* is the threshold for determining algorithm convergence for the number of iterations of *val_step*. Fig 9 presents some image samples of the Arboviruses data sets converted into image by the IGTD with the parameters presented previously. The images are 6x6, this converter is compact and attempts to use as few pixels as possible.

### Results of the Basic CNNs

The Basic CNN was composed of only 1 convolutional layer, 1 max pooling layer, a flatten layer, and 1 dense layer in sequential order. The convolutional layer was configured with 32 filters 3x3, 1 stride, and Rectified Linear Unit (ReLu) as activation function, and because the images are already small, and the convolutional layer further decreases image size, zero padding was added so that the image size remained the same.

We arrived at the Basic CNN architecture empirically, through a series of manual experiments, we explored different parameter combinations, including the number of filters, filter size, stride, neurons, and activation functions. These experiments led us to identify the basic architecture that consistently achieved the highest validation accuracy across all converters.

The max pooling layer was configured with a 2x2 pool size, 1 stride, and zero padding to maintain the image size. The flatten layer does not take any arguments, and the dense layer is configured with 2 neurons, and sigmoid activation function, this model uses the Adam optimizer [39], and cross-entropy for the loss function. As for the ML model, we utilized the best parameters reported by Neto et al. [9] for the XGBoost.

After the conversion of the Arboviruses data, we split each data set into train and test at a ratio of 70% for training and 30% for test, 8013 and 3435 records, respectively, we used the same seed as in Neto et al. [9]. The Basic CNN models, and the XGBoost were trained and tested 30 times, then we calculated the mean of the accuracy and binary average of precision, recall, f1-score, and specificity.

To distinguish the models trained with each converter, we adopted the following naming convention: Basic [Converter name]-CNN. For example, the Basic CNN trained with images generated by DeepInsight is labeled as Basic DeepInsight-CNN, this is consistent across all models.

**Fig 10:**
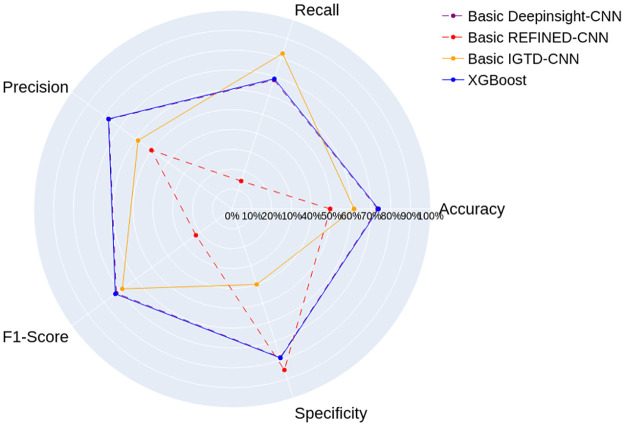
Results of the Basic CNN models and the XGBoost proposed by Neto et al. [9] for the Arboviruses/Inconclusive data set.

The results of the Arbovirus/Inconclusive data set, shown in Fig 10, highlight the Basic REFINED-CNN’s weakest performance across most metrics. The highest specificity of 85%, suggests a better ability to correctly classify Inconclusive cases, yet its weak performance in other metrics makes it unsuitable as the model of choice. In contrast, the Basic IGTD-CNN outperforms the Basic REFINED-CNN in accuracy, recall, precision, and f1-score. Despite the highest recall of 82%, indicating its ability to better identify actual Arbovirus cases, its performance in other metrics still falls bellow the Basic DeepInsight-CNN.

The Basic DeepInsight-CNN stands out by achieving the highest levels of accuracy, precision, and f1-score among all the CNN models. It showcased remarkable competitiveness when compared to the XGBoost, scoring less than 1% bellow in all metrics. It is remarkable that it performs on par with the optimized XGBoost because the Basic DeepInsight-CNN has a simpler architecture of only one convolutional layer, and no hidden dense layers, while the XGBoost is optimized with grid search and feature selection.

Given the small difference between the Basic DeepInsight-CNN and the XGBoost results, we conducted a statistical analysis to determine if this difference is statistically significant. The normality of data was assessed using the Shapiro-Wilk test [40] with a significance level of 0.05. The data samples of all metrics from the Basic DeepInsight-CNN results demonstrated no substantial deviation from a normal distribution. However, the results of the XGBoost exhibited a statistically significant divergence from the normal distribution.

Due to the non-normal distribution of XGBoost’s results, we chose the left-tailed Mann-Whitney U test with significance level 0.05, which does not require a normal distribution to asses the differences between the models [41]. We sought to test whether randomly selected values from the Basic DeepInsight-CNN’s population are likely to be less than those from the XGBoost. Results showed that, for accuracy, the randomly selected values from the Basic DeepInsight-CNN’s results population are statistically expected to be less than those from the XGBoost. However, for recall, precision, f1-score, and specificity, the computed p-values indicates no statistically significant difference between the models.

**Fig 11:**
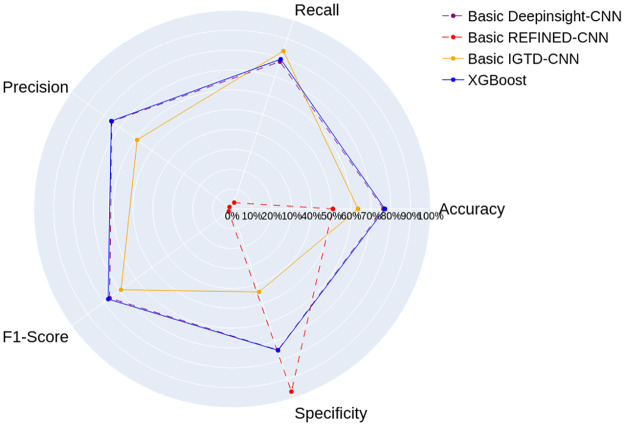
Results of the Basic CNN models and the model proposed by Neto et al. [9] for the Dengue/Chikungunya data set.

Fig 11 shows the results for the Dengue/Chikungunya data set. The Basic REFINED-CNN still has the weakest performance in most metrics, although it achieved the highest specificity value of 96%, the other metrics are under 5% except for accuracy, indicating a possible bias towards Dengue, the negative class in this context. The Basic IGTD-CNN achieves more consistent results, with the highest recall of 85%, but it is still outperformed by the Basic DeepInsight-CNN in the other metrics.

The Basic DeepInsight-CNN stands out once again as the model with the most highest performance metrics among the CNNs. It achieved the highest accuracy, precision, and f1-score among the CNN models. When compared against the XGBoost the Basic DeepInsight-CNN performed about 1% lower in all metrics.

The Shapiro-Wilk normality test with significance level of 0.05 showed that the distribution of the XGBoost results differs from a normal distribution. Therefore, we performed the left-tailed Mann-Whitney U test and the analysis suggested that, except for accuracy and f1-score, the randomly selected values from the Basic DeepInsight-CNN’s population results are inferred to be greater than or equal to those from XGBoost.

After careful evaluation of different CNN models, we observed that the Basic DeepInsight-CNN consistently outperformed alternative CNNs in most metrics, and demonstrated competitive performance comparable to the well-established XGBoost model for both classificaation problems. This makes the Basic DeepInsight-CNN a promising model and the model of choice for further hyperparameter optimization.

### Hyperparameter optimization for the Basic CNN

Based on results of the Basic CNNs, we observed that the Basic DeepInsight-CNN achieved a better and more consistent performance than the remaining CNNs. In addition to that, the Basic DeepInsight-CNN achieved results close to the XGBoost which is a model optimized by grid search and feature selection [9].

Therefore, we optimized the hyperparameters of the Basic DeepInsight-CNN to analyze if it could improve its results, and we call it Tuned DeepInsight-CNN. We performed the hyperparameter optimization by changing the optimizer, the number of convolutional, max pooling and dense layers, the number of filters and strides for each convolutional layer, and the number of neurons for each dense layer.

The optimization was done by using the random search algorithm from the Keras Tuner library [42]. The random search randomly chooses a set of hyperparameters from a given search space and trains a model for each set. The best model is the one with the best validation Accuracy among those chosen by the algorithm. We used 10% of our training set for validation. The search space was defined as presented in Table 5.

**Table 5:** Hyperparameter search space used in the optimization of the Basic DeepInsight-CNN.

Hyperparameter	Values
Number of convolutional layers	1, 2, 3, 4, 5
Number of filters in the convolution layer	32, 64, 128, 256
Kernel Size	3x3
Strides	1, 3
Activation function	ReLu
Number of max pooling layers	1, 2, 3, 4, 5
Pool size	2x2
Strides	1
Number of dense layers	1, 2, 3, 4, 5
Number of neurons in the dense layers	5, 10
Optimizers	Adam, RMSprop, SGD
Loss	Binary crossentropy
Optimization metric	Accuracy

It was necessary to keep the algorithm running for a number of iterations, we determined 1000 iterations given the limited time and resources. Table 6 presents the best set of hyperparameters for the Tuned DeepInsight-CNN that achieved the highest validation accuracy.

**Table 6:** Hyperparameter configuration of the best model returned by the random search, for each Arbovirus data set.

Data set	Layer	Configuration
Arboviruses/Inconclusive	Input	Size: 7x7
	Convolutional	Filters: 256, kernel size: 3x3, strides: 1, padding: ‘same’, activation: ‘relu’
	Max Pooling	Pool size: 2x2, strides: 1
	Convolutional	Filters: 96, kernel size: 3x3, strides: 1, padding: ‘same’, activation: ‘relu’
	Max Pooling	Pool size: 2x2, strides: 1
	Flatten	-
	Dense	Neurons: 256, activation: ‘relu’
	Dense	Neurons: 110, activation: ‘relu’
	Dense	Neurons: 60, activation: ‘relu’
	Dense	Neurons: 2, activation: ‘sigmoid’
Dengue/Chikungunya	Input	Size 7x7
	Convolutional	Filters: 224, kernel size: 3x3, strides: 1, padding: ‘same’, activation: ‘relu‘
	Max Pooling	Pool size: 2x2, strides: 1
	Convolutional	Filters: 256, kernel size: 3x3, strides: 1, padding: ‘same’, activation: ‘relu’
	Max Pooling	Pool size: 2x2, strides: 1
	Convolutional	Filters: 128, kernel size: 3x3, strides: 1, padding: ‘same’, activation: ‘relu’
	Max Pooling	Pool size: 2x2, strides: 1
	Flatten	-
	Dense	Neurons: 210, activation: ‘relu’
	Dense	Neurons: 160, activation: ‘relu’
	Dense	Neurons: 110, activation: ‘relu’
	Dense	Neurons: 2, activation: ‘sigmoid’

**Fig 12:**
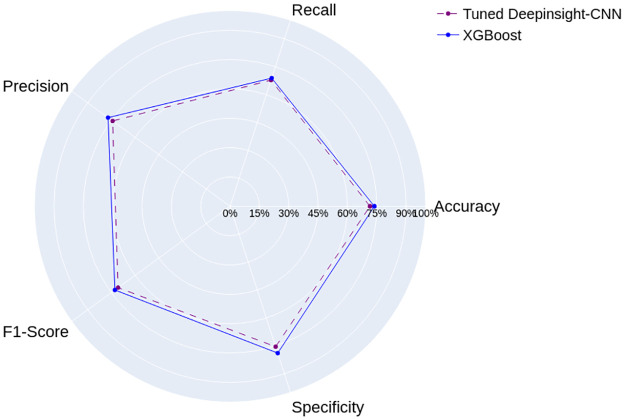
Results of the Tuned DeepInsight-CNN and the model proposed by Neto et al. [9], for the Arboviruses/Inconclusive data set.

Fig 12 shows the results of the Tuned DeepInsight-CNN, trained on images from the Arboviruses/Inconclusive data set, and the XGBoost, proposed by Neto et al. [9]. In general, both models demonstrated similar performances. The XGBoost achieved only 2% improvement on average across the metrics. As mentioned before, the XGBoost’s results do not follow a normal distribution. So we applied the left-tailed Mann-Whitney U test with significance level 0.05, to analyze the differences between the models. We found that, except for specificity, the randomly selected values from the Tuned DeepInsight-CNN’s results population are consistently anticipated to be lower than those from the XGBoost’s population.

Although the Tuned DeepInsight-CNN has a more complex architecture, it demonstrated consistent results without any apparent indications of bias towards a particular class. However, the hyperparameter optimization did not improve its results significantly compared to its Basic version.

**Fig 13:**
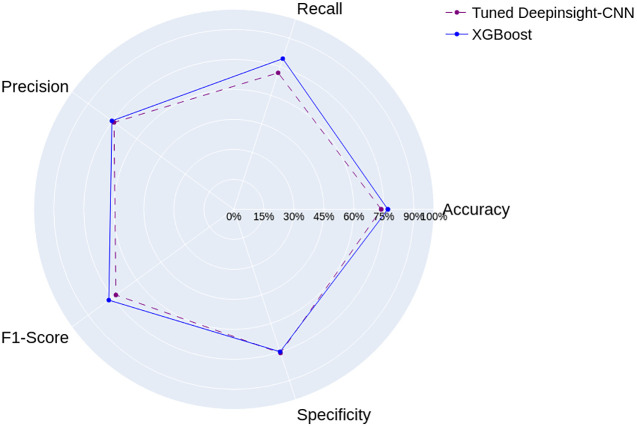
Results of the Tuned DeepInsight-CNN and the model proposed by Neto et al. [9], for the Dengue/Chikungunya data set.

The results of the Tuned DeepInsight-CNN for the Dengue/Chikungunya data are presented in Fig 13. The results demonstrate that the XGBoost outperformed the Tuned DeepInsight-CNN across most metrics by about 2.4% on average, except for specificity.

Similar to the previous classification problem, the left-tailed Mann-Whitney U test with significance level 0.05 was applied and it showed that the Tuned DeepInsight-CNN’s results are statistically lower than those from the XGBoost’s results population, except for specificity. These findings indicate that the XGBoost is more effective at accurately classifying both Dengue and Chikungunya cases in this particular context.

Our experiments demonstrated that the Tuned DeepInsight-CNN achieved close results with respect to the XGBoost, for both data sets. The hyperparameter optimization did not improve its performances significantly, and was not enough to outperform the XGBoost. However, the performance of the Tuned DeepInsight-CNN could be further improved with more iterations over the search space, or a different one. Overall, the results highlight the potential of both models, for the classification of Arboviruses.

## Discussion

In this work, we demonstrated that even a Basic CNN trained on images generated from the conversion of tabular data can perform competitively to a tree-based ML algorithm, the XGBoost, that was trained and optimized on the original data. The results of the Basic DeepInsight CNN were found to be statistically greater than or equal to those of the XGBoost for most metrics (except for accuracy for the Arbovirus/Inconclusive data set, and accuracy and f1-score for the Dengue/Chikungunya data set).

In an attempt to improve the performance of the Basic CNN, and hopefully outperform the XGBoost significantly, we applied the hyperparameter optimization technique random search. We optimized the CNN trained on images from the converter that resulted in the highest metrics for the basic models, the DeepInsight, resulting in the Tuned DeepInsight-CNN. However, after optimization, results showed no significant improvement in performance compared to both the Basic CNN and the XGBoost. Additionally, except for specificity, the Tuned DeepInsight-CNN’s results were statistically lower according to the left-tailed Mann-Whitney U test.

Neither the CNN nor the XGBoost reached metrics over 80%, which can be attributed to several factors. One of which is the nature of the data set. Challenges intrinsic to the diagnosis of Arboviruses may have contributed negatively to the performances of the models. As mentioned before, the diagnosis of Arboviruses is complex, the diseases in question share symptoms and can be indistinguishable from each other in some cases [27]. In Addition to that, co-infection is common and can further increase the difficulty of the clinical diagnosis [27]. Since the data set is composed of cases confirmed by clinical diagnosis, the difficulties in diagnosis are reflected in the data. As a result, the data set may contain inaccurate data, this can potentially impact the models’ performance.

The use of converters for tabular data into images encode feature distance information as 2D spacial arrangement, which can be picked up by the CNN and improve its performance [18, 19]. As mentioned previously in Related works, experiments performed by the creators of the converters, demonstrated that the conversion of tabular data into images to train CNNs can outperform ML algorithms in several data sets [18, 19]. We expected a substantial increase in performance of the CNNs with respect to the XGBoost, specially after hyperparameter optimization. However, we found no statistically significant difference in the highest performing Basic CNN, and statistically lower results of its tuned version compared to the XGBoost.

The lack of a substantial improvement after the conversion to images, despite the added feature distance information as 2D spatial distance, may indicate that this approach did not effectively provide additional information to enable the CNN to gain a robust understanding of the features in the data. In other words, the visual representation of the data, albeit enhanced with spatial information, may not have sufficiently equipped the CNN to grasp the underlying patterns in the data with adequate accuracy.

The hyperparameter optimization still did not result in a CNN model that outperformed the XGBoost significantly. However, training for more iterations, or modifying the search space could improve the performances of the CNNs. Moreover, making use of different transfer learning techniques, and ensemble methods that integrate the strengths of both approaches may yield even more robust classification models

The challenges posed by the classification of Arboviruses diseases, combined with the limitations of the data representation and potentially the size of the data set, could be potential factors that hindered the improvement in performance with the use of a CNN.

## Conclusion

In this work, we conducted a comparative analysis of converters for tabular data into images. First, we enumerated and studied the converters of tabular data into images, exploring 3 different approaches available in the literature. We proposed a methodology to select the best converter, based on the evaluation of Basic CNN models. Lastly, we optimized a Tuned CNN trained on images from the best converter, and compared the results with the ML algorithm XGBoost. Results demonstrated the potential of using image-based approaches for the classification of Arboviruses.

We demonstrated that even a Basic CNN model can achieve metrics comparable to an XGBoost model optimized by grid search and feature selection trained on the original tabular data. We also noticed that the optimization of the CNN, with random search with the given search space, did not improve its performance significantly.

As a work limitation, we mention that this is a new research field, the literature provides a limited number of converters for tabular data into image, and our work only analyzed 3 of them. Another limitation is that this work studied the use of 2D CNNs only, trained on images converted from tabular data. Although it is possible to use 1D CNN models on the tabular data in original form, we did not use it. We limited the scope to 2D CNNs because of the possible performance gain from the 2D spacial information.

One aspect to consider, with respect to the comparison, is the number of features used by each model. Our CNN models used all the 27 features present in the preprocessed data set. While the ML model that reached the highest metrics presented by Da Silva Neto et al. [9], the XGBoost, used 17 and 22 features for the Arbovirus/Inconclusive and Dengue/Chikungunya data sets, respectively, chosen by a feature selection algorithm. The feature selection may have been an advantage for the XGBoost.

As future works, we plan to further study the optimization of CNN architectures for image representations of tabular data, explore alternative converters for tabular data into images, the use of 1D CNN, and transfer learning techniques. Additionally, future works may extend this methodology to other health data sets and analyze the performance of models. We also plan to include computational cost analysis to evaluate CNN efficiency compared to traditional ML models with respect to resource consumption.

Future research could also explore the realm of Explainable Artificial Intelligence (XAI) to enhance the interpretability of CNN for disease classification with images generated from tabular data. Lastly, to analyze the generalizability of the models for the classification of Arboviruses, future research could also include regional and temporal data slicing.

In summary, transforming tabular data into images to leverage the power of CNNs has the potential to increase the model performance by the additional 2D spatial information that can be processed by the CNN. Nonetheless, in instances where the data set naturally presents challenges in classification, the use of CNNs might not be sufficient to improve the performance significantly.

We concluded our general objective of comparing the effectiveness of different converters from the current literature in transforming tabular data into images. It’s important to note that these results are specific to the data set and the experiments presented here, and may not generalize to other data sets. Nonetheless, this study provides valuable insights into the performance of CNNs trained on images converted from tabular data, and highlights the potential benefits and limitations of using image-based DL models for this type of data.

## Abbreviations

AI: Artificial Intelligence
BMDS: Bayesian Multidimensional Scaling
CNN: Convolutional Neural Network
CQA: Conversion Quality Analysis
DL: Deep Learning
DR: Dimensionality Reduction
DT: Decision Tree
FDM: Feature Density Matrix
FN: False Negative
FP: False Positive
IGTD: Image Generator for Tabular Data
kPCA: Kernel Principal Component Analysis
MDS: Multidimensional Scaling
ML: Machine Learning
OF: Percentage of Overlapped Features
OP: Percentage of Overlapped Pixels
PCA: Principal Component Analysis
REFINED: REpresentation of Features as Images with NEighborhood Dependencies
ReLu: Rectified Linear Unit
SINAN: Brazilian Information System for Notifiable Diseases
TN: True Negative
TP: True Positive
T-SNE: T-Distributed Stochastic Neighbor Embedding
XAI: Explainable Artificial Intelligence
XGBooste: Xtreme Gradient Boosting
